# Supplementary material for: Affordability of Food and Beverages in Mexico between 1994 and 2016
Source: Nutrients. 2019 Jan 3;11(1):78. doi: 10.3390/nu11010078 (PMC6356212; doi:10.3390/nu11010078)
Supplement: Supplementary file 1 [file nutrients-11-00078-s001.pdf]

Table S1. List of foods purchased by energy quintiles Mexico, ENIGH 1994–2016

Quintile	Food item
I	Radish, chard, spinach, pumpkin, onion, chayote, jalapeño pepper, serrano pepper, cilantro, cabbage, epazote, tomato, lettuce, cactus, cucumber, green tomato, other vegetables, packaged chiles, cherry, raspberry, strawberry, blackberry, lime, lemon, papaya, pineapple, fresh mushrooms.
II	Beef steak, flank steak, pork intestines, pork ham, chicken entrails, whole fish, fresh shrimp, fresh seafood, other milk derivatives, beets, potatoes, other tubers, broccoli, poblano chili, green beans, corn, parsley, peppermint, carrots, vegetables and pulses, frozen vegetables and legumes, processed beans, soursop, apricot, peach, chicozapote, plum guava, tangerine, grapefruit, mango, apple, orange, pear, prickly pear, banana, other bananas, grape, other fruits, fruits in syrup, mustard, sweet and spicy sauces, baby food.
III	Corn dough, beef chamorro, beef steak, special cuts of beef, beef burger for grilling, ground beef, meat from other parts of beef, pork steak, pork leg, sausage, leg chicken, chicken sausage, goat, other meats, fish fillet, canned tuna, eel, cream, chicken egg, avocado, garlic, pea, other chili peppers, other legumes, plantain, mole in pasta, other prepared foods, ice creams, snows and ice pops
IV	Wheat flour, flour tortilla, white bread, bread for sandwich, cakes and pastries, other wheat products, rice grain, beef milanesa, beef stew, piece of beef pulp, beef intestines, pork pulp, ground pork, pork rib, pork shoulder, meat from other parts of the pork, beef enchilada, mashed beef and dried meat, mortadella, other processed meats, whole chicken, other poultry, salmon, cod, smoked fish, processed seafood, fresh cheese, manchego cheese, Oaxaca cheese, other cheeses, flour for mashed potatoes, dried chilies, beans, beans, lentils, crystallized fruits, other chocolates, cinnamon, cloves, pepper, breakfast cereal, prepared pizzas, carnitas, roasted chicken, barbecue, insects, custards, jellies, cajeta, "dulce de leche", peanut butter.
V	Corn in grain, corn flour, toast, sweet biscuits, crackers, sweet bread, other rice products, corn cereal, snacks, instant soups, other cereals, beef rib chop, pork rinds, chorizo, smoked pork chop pork, bacon, yellow cheese, aged cheese, Chihuahua cheese, butter, vegetable oil, coconut oil, margarine, pork lard, shortening, other oils, potato chips, corn sprouts, seeds in bulk, seeds, seeds processed, other dressings, other treats.

Table S2. List of food and beverages by quintile of Nutrient Rich Food Index, Mexico, ENIGH 1994–2016

Quintile	Food or beverage item
I	Sweet bread, cakes and pastries, beef rib chop, pork steak, ground pork, pork knuckle, chorizo, mortadella, bacon, sausages, other processed meats, condensed milk, aged cheese, other cheeses, butter, others milk derivatives, coconut oil, pork lard, shortening, grape, crystallized fruit, chocolate tablet, other dressings, puddings, jellies, cajeta, “dulce de leche”, peanut butter, ice cream, ice cream, ice pops, other sweets, mineral water, cola and flavored drinks, energy drinks, fermented corn drinks, liquor or fruit creams.
II	Flour tortilla, sweet biscuits, crackers, white bread, snacks, beef milanesa, grilled beef, beef stew, special cuts of beef, pork rib, enchilada meat, pork rinds, smoked pork chop, pork ham, whole chicken, chicken sausage, fish fillet, pasteurized cow's milk, unpasteurized milk, yellow cheese, Chihuahua cheese, vegetable oil, margarine, potato chips, corn germinated, packaged chilli, fruits in syrup, pizzas prepared, cognac, brandy, beer, brandy, rum, tequila, table wine, vodka, whiskey, other alcoholic beverages.
III	Corn in grain, corn flour, corn dough, corn tortilla, other corn products, wheat flour, soup paste, sandwich bread, rice grain, other rice products, beef steak, beef shanks for roast, ground beef, meat from other parts of beef, pork pulp, pork shoulder, meat from other pork farms, machaca and dried meat, leg of chicken, other poultry, goat, lamb, fresh shrimp, evaporated milk , modified milk, other milk, fresh cheese, Manchego cheese, oaxaca cheese, fermented milk drinks, chicken eggs, other eggs, flour for mashed potatoes, seeds in bulk, packaged seeds, apple, pear, green banana, other bananas , other fruits, mole in pasta, mustard, roasted chicken, barbecue, other prepared foods, mead, eggnog.
IV	Corn cereal, instant soups, other cereals, flank steak, beef chamorro, beef pulp, pork leg, other meats, whole fish, canned tuna, eels, salmon, smoked fish, fresh seafood, processed seafood, whole milk powder , potatoes, other tubers, avocado, garlic, onion, chayote, pea, corn, cucumber, vegetables and legumes, grain beans, chickpea beans, beans, lentils, other legumes in grain, processed beans, other processed legumes, processed seeds , guanabana, apricot, peach, chicozapote, mamey, plum, tangerine, negarine, tangerine, mango, pineapple, banana, watermelon, roasted coffee, chocolate powder, clove, pepper, sweet and spicy sauces, rice cereal, juices fruits and vegetables for baby, fresh mushrooms, insects, prepared water and natural juices, juices and bottled nectars.
V	Other wheat products, pork intestines, chicken intestines, beets, radishes, Swiss chard, spinach, broccoli, pumpkin, jalapeño pepper, chile poblano, chile serrano, other peppers, cilantro, cabbage, green beans, epazote, tomato, milk, cactus , parsley, peppermint, tomato, carrot, other vegetables, dried chiles, cherry, raspberry, guava, lemon, lime, grapefruit, melon, orange, papaya, tuna, other chocolates, concentrates and other powders to prepare drinks.

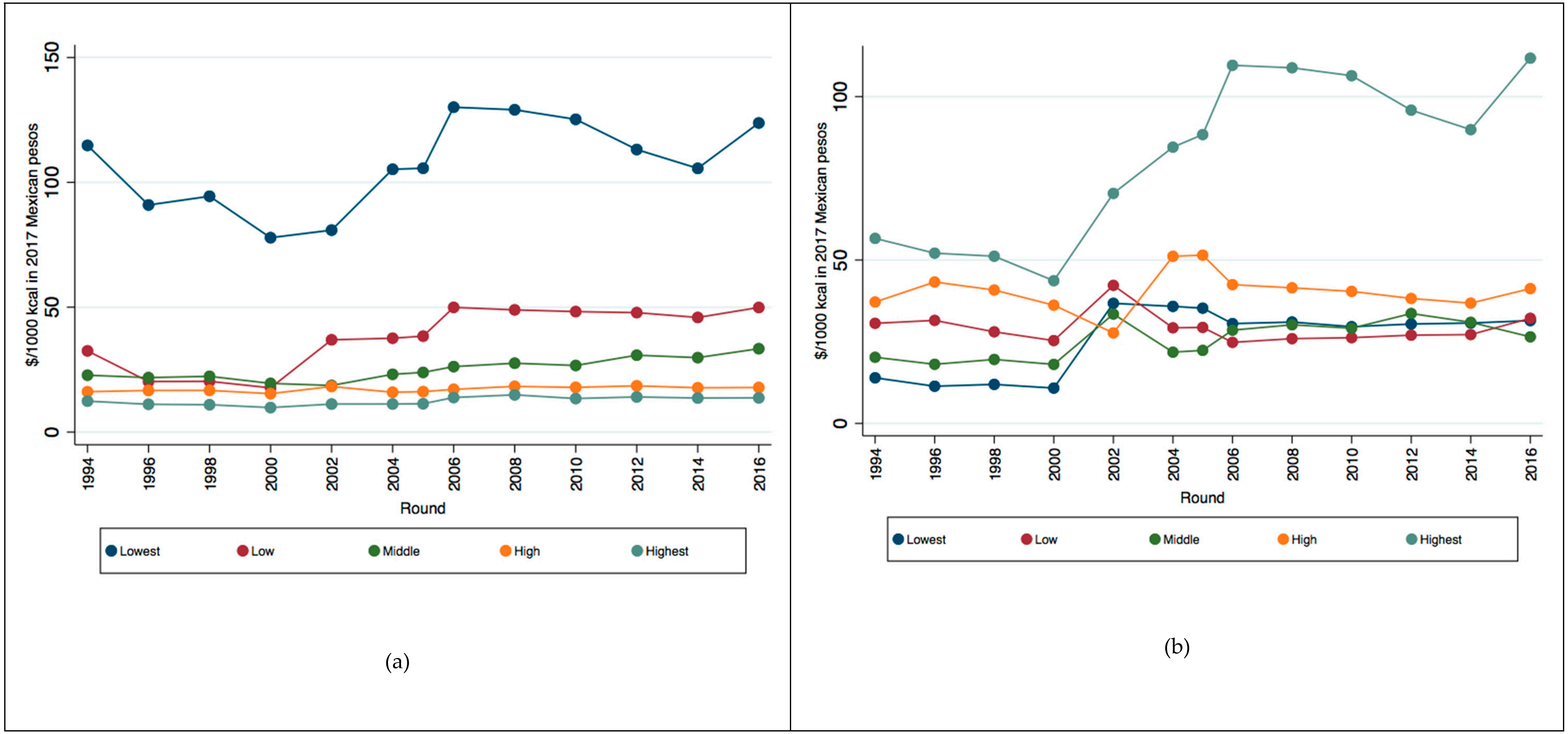

Figure S1. Costs per 1,000 kcal of food and beverages, 1994–2016. (a) Cost per 1000 kcal by quintile of energy density. (b) Cost per 1000 kcal by quintile of Nutrient Rich Food Index. Authors estimations using the National Income and Expenditure Survey (1994–2016). Quintiles of energy density and NRFI were calculated on the 222 categories (for energy density excluding all beverages categories).

Table S3- Descriptive statistics of 68,517 households in 2016, ENIGH

Variable		%
Male head of the household		72.4
Household member are males		48.3
Children under 12 years old		17.1
Urban		78.3
Education of the head of the household		
	Primary school or less	43.4
	Secondary school	29.1
	Highschool	14.5
	College or more	12.3
Household size household (mean)		3.7
